# Hexa­kis­(μ_2_-4-amino-3,5-dimethyl-4*H*-1,2,4-triazole)hexa­aqua­tricobalt(II) naphthalene-1,5-disulfonate tetra­chloride

**DOI:** 10.1107/S1600536812020417

**Published:** 2012-06-02

**Authors:** Zhi-Guo Gu, Bao-Xiang Wang, Chun-Yan Pang, Fei-Fei Bao

**Affiliations:** a School of Chemical and Material Engineering, Jiangnan University, Wuxi 214122, People’s Republic of China

## Abstract

In the centrosymmetric trinuclear cation of the title compound, [Co_3_(C_4_H_8_N_4_)_6_(H_2_O)_6_](C_10_H_6_O_6_S_2_)Cl_4_, the three Co^II^ atoms are bridged by six triazole mol­ecules *via* the N atoms in the 1,2-positions. The central Co^II^ atom, lying on an inversion center, is coordinated by six triazole N atoms while the terminal Co^II^ atoms are coordinated by three triazole N atoms and three water O atoms in a distorted octa­hedral geometry. The naphthalene­disulfonate anion is also centrosymmetric. The four chloride counter anions are half-occupied; the H atoms of the amino groups show an occupancy of 2/3. O—H⋯Cl, O—H⋯O and N—H⋯O hydrogen bonds link the complex cations and the chloride and naphthalene-1,5-disulfonate anions.

## Related literature

For the structure of the title cation as hydrated nitrate salt, see: Tong *et al.* (2011 ▶).
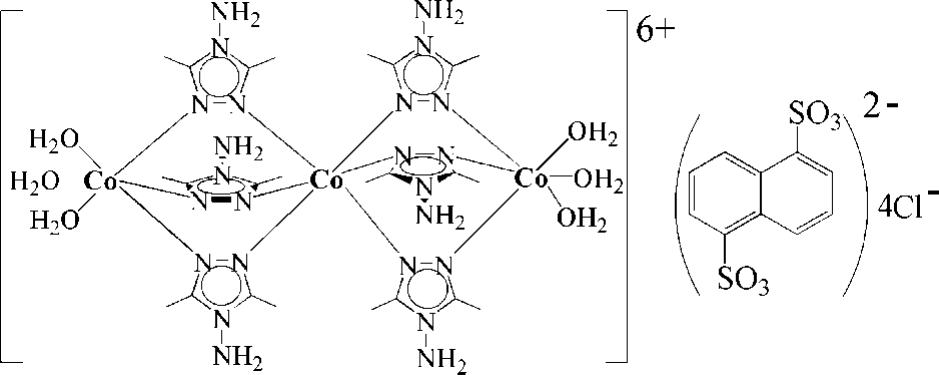


## Experimental

### 

#### Crystal data

[Co_3_(C_4_H_8_N_4_)_6_(H_2_O)_6_](C_10_H_6_O_6_S_2_)Cl_4_
*M* *_r_* = 1385.82
Triclinic, 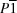

*a* = 11.1641 (4) Å
*b* = 12.2744 (5) Å
*c* = 13.2265 (5) Å
α = 106.924 (2)°
β = 99.622 (3)°
γ = 103.452 (2)°
*V* = 1631.79 (11) Å^3^
*Z* = 1
Mo *K*α radiation
μ = 1.05 mm^−1^
*T* = 100 K
0.26 × 0.22 × 0.20 mm

#### Data collection

Bruker SMART APEX CCD diffractometer
Absorption correction: multi-scan (*SADABS*; Bruker, 2001 ▶) *T*_min_ = 0.763, *T*_max_ = 0.815
23481 measured reflections
6410 independent reflections
5057 reflections with *I* > 2σ(*I*)
*R*_int_ = 0.043

#### Refinement

*R*[*F* ^2^ > 2σ(*F* ^2^)] = 0.048
*wR*(*F* ^2^) = 0.108
*S* = 1.06
6410 reflections
386 parameters
H-atom parameters constrained
Δρ_max_ = 0.67 e Å^−3^
Δρ_min_ = −0.75 e Å^−3^

### 

Data collection: *SMART* (Bruker, 2007 ▶); cell refinement: *SAINT* (Bruker, 2007 ▶); data reduction: *SAINT*; program(s) used to solve structure: *SHELXTL* (Sheldrick, 2008 ▶); program(s) used to refine structure: *SHELXTL*; molecular graphics: *DIAMOND* (Brandenburg, 1999 ▶); software used to prepare material for publication: *SHELXTL*.

## Supplementary Material

Crystal structure: contains datablock(s) I, global. DOI: 10.1107/S1600536812020417/hy2535sup1.cif

Structure factors: contains datablock(s) I. DOI: 10.1107/S1600536812020417/hy2535Isup2.hkl

Additional supplementary materials:  crystallographic information; 3D view; checkCIF report

## Figures and Tables

**Table 1:** Hydrogen-bond geometry (Å, °)

*D*—H⋯*A*	*D*—H	H⋯*A*	*D*⋯*A*	*D*—H⋯*A*
O1*W*—H1*X*⋯Cl3^i^	0.98	2.06	2.765 (3)	127
O1*W*—H1*Y*⋯Cl3^ii^	0.98	1.89	2.701 (3)	138
O2*W*—H2*X*⋯Cl1	0.98	2.28	2.929 (3)	122
O2*W*—H2*Y*⋯Cl3^ii^	0.98	2.82	3.749 (3)	158
O3*W*—H3*X*⋯O2^iii^	0.98	1.89	2.773 (3)	148
O3*W*—H3*Y*⋯Cl4^iii^	0.98	2.18	3.114 (3)	159
N8—H8*A*⋯O3^i^	0.91	2.21	3.009 (3)	146

Symmetry codes: (i) 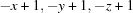; (ii) 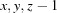; (iii) 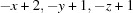.

**Fig. 1.**
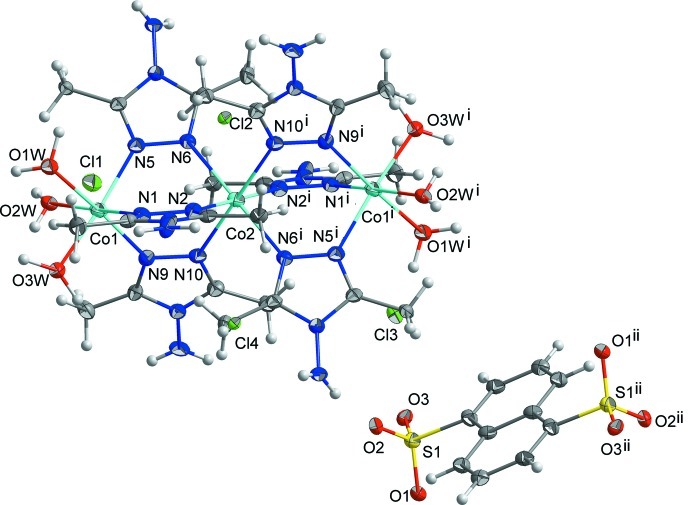
The molecular structure of the title compound. Displacement ellipsoids are drawn at the 50% probability level. [Symmetry codes: (i) 1-x, 1-y, 1-z; (ii) 2-x, -y, 2-z.]

## supplementary crystallographic information

### Comment

A recent study has reported a centrosymmetric trinuclear cobalt nitrate complex of 4-amino-3,5-dimethanyl-1,2,4-triazole (C_4_H_8_N_4_), which crystallizes in the monoclinic system, *P*2_1_/n space group (Tong *et al.*, 2011). The charge of the hexavalent cation, [Co_3_(C_4_H_8_N_4_)_6_(H_2_O)_6_]^6+^, is balanced by the nitrate anions. In the trinuclear unit of the title compound, the central Co^II^ atom and two terminal Co^II^ atoms are bridged by six triazole ligands (Fig. 1). The central Co^II^ atom, lying on an inversion center, is coordinated by six N atoms from the triazole ligands whereas each terminal Co^II^ atom completes its octahedral geometry by three water molecules. The title compound displays a similar structure to the previuosly reported cobalt analogs (Tong *et al.*, 2011), but it crystallizes in the triclinic system, *P*1 space group. The charge of the trinuclear cation is balanced by one naphthalene-1,5-disulfonate dianion and four chloride anions. O—H···Cl, O—H···O and N—H···O hydrogen bonds link the complex cations, chloride and naphthalene-1,5-disulfonate anions (Table 1).

### Experimental

A 20 ml ethanol solution of 4-amino-3,5-dimethanyl-1,2,4-triazole (0.6 mmol) was mixed with a 10 ml aqueous solution of CoCl_2_.6H_2_O (0.3 mmol). naphthalene-1,5-disulfonate acid (0.1 mmol) in 5 ml of ethanol was added to this reaction mixture with continuous stirring. The resulting solution was filtered and left to stand at room temperature. Pink bolck-shaped crystals of the title compound were obtained by slow evaporation of the solvent within two weeks (yield: 48%).

### Refinement

H atoms on water molecules and on N8 atom were located from a difference Fourier map and refined as riding atoms, with *U*_iso_(H) = 1.2*U*_eq_(O) or 1.5*U*_eq_(N). The other H atoms were positioned geometrically and refined as riding atoms, with C—H = 0.95 (CH) and 0.98 (CH_3_) and N—H = 0.88 Å and with *U*_iso_(H) = 1.2(1.5 for methyl)*U*_eq_(C, N). Cl atoms were refined with a fixed occupancy factor of 0.50.

### Crystal data

**Table:**

[Co_3_(C_4_H_8_N_4_)_6_(H_2_O)_6_](C_10_H_6_O_6_S_2_)Cl_4_	*Z* = 1
*M**_r_* = 1385.82	*F*(000) = 715
Triclinic, *P*1	*D*_x_ = 1.410 Mg m^−^^3^
Hall symbol: -P 1	Mo *K*α radiation, λ = 0.71073 Å
*a* = 11.1641 (4) Å	Cell parameters from 3738 reflections
*b* = 12.2744 (5) Å	θ = 2.2–25.7°
*c* = 13.2265 (5) Å	µ = 1.05 mm^−^^1^
α = 106.924 (2)°	*T* = 100 K
β = 99.622 (3)°	Block, pink
γ = 103.452 (2)°	0.26 × 0.22 × 0.20 mm
*V* = 1631.79 (11) Å^3^	

### Data collection

**Table:**

Bruker SMART APEX CCD diffractometer	6410 independent reflections
Radiation source: sealed tube	5057 reflections with *I* > 2σ(*I*)
Graphite monochromator	*R*_int_ = 0.043
φ and ω scans	θ_max_ = 26.0°, θ_min_ = 1.7°
Absorption correction: multi-scan (*SADABS*; Bruker, 2001)	*h* = −13→13
*T*_min_ = 0.763, *T*_max_ = 0.815	*k* = −15→15
23481 measured reflections	*l* = −16→16

### Refinement

**Table:**

Refinement on *F*^2^	Secondary atom site location: difference Fourier map
Least-squares matrix: full	Hydrogen site location: inferred from neighbouring sites
*R*[*F*^2^ > 2σ(*F*^2^)] = 0.048	H-atom parameters constrained
*wR*(*F*^2^) = 0.108	*w* = 1/[σ^2^(*F*_o_^2^) + (0.0427*P*)^2^ + 2.0543*P*] where *P* = (*F*_o_^2^ + 2*F*_c_^2^)/3
*S* = 1.06	(Δ/σ)_max_ = 0.001
6410 reflections	Δρ_max_ = 0.67 e Å^−^^3^
386 parameters	Δρ_min_ = −0.75 e Å^−^^3^
0 restraints	Extinction correction: *SHELXTL* (Sheldrick, 2008), Fc^*^=kFc[1+0.001xFc^2^λ^3^/sin(2θ)]^-1/4^
Primary atom site location: structure-invariant direct methods	Extinction coefficient: 0.0020 (5)

### Special details

**Table:**

Geometry. All esds (except the esd in the dihedral angle between two l.s. planes) are estimated using the full covariance matrix. The cell esds are taken into account individually in the estimation of esds in distances, angles and torsion angles; correlations between esds in cell parameters are only used when they are defined by crystal symmetry. An approximate (isotropic) treatment of cell esds is used for estimating esds involving l.s. planes.
Refinement. Refinement of *F*^2^ against ALL reflections. The weighted *R*-factor *wR* and goodness of fit *S* are based on *F*^2^, conventional *R*-factors *R* are based on *F*, with *F* set to zero for negative *F*^2^. The threshold expression of *F*^2^ > σ(*F*^2^) is used only for calculating *R*-factors(gt) *etc*. and is not relevant to the choice of reflections for refinement. *R*-factors based on *F*^2^ are statistically about twice as large as those based on *F*, and *R*- factors based on ALL data will be even larger.

### Fractional atomic coordinates and isotropic or equivalent isotropic displacement parameters (Å^2^)

**Table:**

	*x*	*y*	*z*	*U*_iso_*/*U*_eq_	Occ. (<1)
Co1	0.63106 (4)	0.64428 (4)	0.31065 (4)	0.02470 (13)	
Co2	0.5000	0.5000	0.5000	0.02126 (15)	
O1W	0.5966 (2)	0.56654 (19)	0.14192 (17)	0.0265 (5)	
H1X	0.5055	0.5253	0.1106	0.040*	
H1Y	0.6234	0.6285	0.1105	0.040*	
O2W	0.6118 (2)	0.79286 (19)	0.27619 (18)	0.0261 (5)	
H2X	0.6232	0.8578	0.3444	0.039*	
H2Y	0.5942	0.7753	0.1971	0.039*	
O3W	0.8229 (2)	0.70517 (19)	0.31226 (18)	0.0268 (5)	
H3X	0.8292	0.7098	0.2406	0.040*	
H3Y	0.8764	0.7196	0.3845	0.040*	
N1	0.6573 (2)	0.4793 (2)	0.3235 (2)	0.0223 (6)	
N2	0.6086 (2)	0.4269 (2)	0.3951 (2)	0.0215 (5)	
N3	0.7090 (2)	0.3163 (2)	0.3066 (2)	0.0213 (5)	
N4	0.7638 (3)	0.2234 (2)	0.2677 (2)	0.0229 (6)	
H4A	0.7329	0.1885	0.1940	0.034*	0.667
H4B	0.7436	0.1680	0.3001	0.034*	0.667
H4C	0.8499	0.2545	0.2842	0.034*	0.667
N5	0.4307 (2)	0.5840 (2)	0.2976 (2)	0.0223 (6)	
N6	0.3797 (2)	0.5345 (2)	0.3702 (2)	0.0210 (5)	
N7	0.2246 (2)	0.5399 (2)	0.2502 (2)	0.0214 (5)	
N8	0.0988 (2)	0.5242 (2)	0.1957 (2)	0.0233 (6)	
H8A	0.0997	0.5532	0.1398	0.035*	0.667
H8B	0.0604	0.5640	0.2434	0.035*	0.667
H8C	0.0549	0.4451	0.1687	0.035*	0.667
N9	0.6756 (2)	0.7319 (2)	0.4850 (2)	0.0234 (6)	
N10	0.6331 (2)	0.6759 (2)	0.5555 (2)	0.0214 (5)	
N11	0.7398 (2)	0.8597 (2)	0.6501 (2)	0.0228 (6)	
N12	0.7993 (3)	0.9696 (2)	0.7368 (2)	0.0302 (7)	
H12A	0.8319	1.0268	0.7095	0.045*	0.667
H12B	0.8632	0.9615	0.7839	0.045*	0.667
H12C	0.7411	0.9911	0.7727	0.045*	0.667
C1	0.7852 (3)	0.4237 (3)	0.1853 (3)	0.0292 (7)	
H1D	0.8529	0.5001	0.2143	0.044*	
H1E	0.7246	0.4222	0.1217	0.044*	
H1F	0.8225	0.3584	0.1635	0.044*	
C2	0.7179 (3)	0.4095 (3)	0.2710 (2)	0.0213 (6)	
C3	0.6427 (3)	0.3295 (3)	0.3836 (2)	0.0204 (6)	
C4	0.6189 (3)	0.2409 (3)	0.4412 (2)	0.0206 (6)	
H4D	0.5658	0.2627	0.4911	0.031*	
H4E	0.7003	0.2410	0.4829	0.031*	
H4F	0.5751	0.1611	0.3874	0.031*	
C5	0.3332 (3)	0.6312 (3)	0.1326 (3)	0.0209 (6)	
H5D	0.4186	0.6815	0.1396	0.031*	
H5E	0.2731	0.6780	0.1321	0.031*	
H5F	0.3068	0.5630	0.0642	0.031*	
C6	0.3351 (3)	0.5871 (3)	0.2273 (2)	0.0219 (6)	
C7	0.2558 (3)	0.5085 (3)	0.3398 (2)	0.0219 (6)	
C8	0.1563 (3)	0.4586 (3)	0.3906 (3)	0.0287 (7)	
H8D	0.1968	0.4413	0.4533	0.043*	
H8E	0.0959	0.3850	0.3367	0.043*	
H8F	0.1110	0.5169	0.4150	0.043*	
C9	0.8092 (3)	0.9393 (3)	0.5089 (3)	0.0281 (7)	
H9A	0.7569	0.9922	0.5030	0.042*	
H9B	0.8251	0.9038	0.4377	0.042*	
H9C	0.8905	0.9853	0.5627	0.042*	
C10	0.7414 (3)	0.8433 (3)	0.5442 (2)	0.0211 (6)	
C11	0.6736 (3)	0.7529 (3)	0.6545 (2)	0.0231 (7)	
C12	0.6622 (3)	0.7396 (3)	0.7613 (3)	0.0245 (7)	
H12D	0.6123	0.6577	0.7490	0.037*	
H12E	0.6196	0.7953	0.7974	0.037*	
H12F	0.7473	0.7569	0.8079	0.037*	
S1	1.06020 (7)	0.26397 (7)	0.94747 (6)	0.02274 (18)	
O1	1.1780 (2)	0.32512 (18)	1.03387 (17)	0.0229 (5)	
O2	1.0829 (2)	0.21065 (18)	0.84071 (17)	0.0222 (5)	
O3	0.9768 (2)	0.33792 (18)	0.94574 (17)	0.0238 (5)	
C13	0.7946 (3)	0.0438 (3)	1.0283 (2)	0.0211 (6)	
H13	0.7140	0.0431	1.0427	0.025*	
C14	0.8632 (3)	0.1383 (3)	1.0024 (3)	0.0235 (7)	
H14	0.8283	0.2011	0.9992	0.028*	
C15	0.9796 (3)	0.1417 (3)	0.9818 (3)	0.0229 (7)	
C16	1.0358 (3)	0.0482 (3)	0.9874 (3)	0.0226 (6)	
C17	1.1565 (3)	0.0484 (3)	0.9670 (3)	0.0245 (7)	
H17	1.2041	0.1126	0.9501	0.029*	
Cl1	0.45668 (16)	0.91733 (15)	0.39605 (14)	0.0331 (4)	0.50
Cl2	0.47349 (13)	0.01196 (12)	0.18426 (11)	0.0180 (3)	0.50
Cl3	0.57922 (14)	0.65370 (13)	0.97649 (13)	0.0249 (3)	0.50
Cl4	0.95318 (12)	0.22467 (12)	0.48318 (11)	0.0172 (3)	0.50

### Atomic displacement parameters (Å^2^)

**Table:**

	*U*^11^	*U*^22^	*U*^33^	*U*^12^	*U*^13^	*U*^23^
Co1	0.0198 (2)	0.0236 (2)	0.0234 (2)	0.00047 (17)	0.00243 (17)	0.00400 (18)
Co2	0.0170 (3)	0.0209 (3)	0.0225 (3)	0.0040 (2)	0.0033 (2)	0.0048 (2)
O1W	0.0259 (12)	0.0256 (11)	0.0212 (11)	0.0007 (9)	0.0044 (9)	0.0047 (9)
O2W	0.0255 (12)	0.0253 (11)	0.0179 (11)	−0.0031 (9)	0.0005 (9)	0.0047 (9)
O3W	0.0229 (12)	0.0313 (12)	0.0218 (11)	0.0013 (9)	0.0037 (9)	0.0094 (10)
N1	0.0169 (13)	0.0225 (13)	0.0235 (14)	0.0027 (10)	0.0053 (11)	0.0043 (11)
N2	0.0226 (13)	0.0186 (12)	0.0208 (13)	0.0064 (10)	0.0067 (11)	0.0024 (10)
N3	0.0245 (13)	0.0222 (13)	0.0171 (13)	0.0120 (11)	0.0064 (11)	0.0022 (10)
N4	0.0327 (15)	0.0256 (14)	0.0203 (13)	0.0210 (12)	0.0139 (11)	0.0085 (11)
N5	0.0223 (14)	0.0188 (13)	0.0214 (14)	0.0036 (10)	0.0026 (11)	0.0039 (10)
N6	0.0180 (13)	0.0211 (13)	0.0213 (13)	0.0045 (10)	0.0047 (10)	0.0047 (11)
N7	0.0188 (13)	0.0228 (13)	0.0201 (13)	0.0043 (10)	0.0037 (10)	0.0059 (11)
N8	0.0166 (13)	0.0243 (13)	0.0256 (14)	0.0034 (10)	−0.0009 (11)	0.0092 (11)
N9	0.0232 (14)	0.0245 (13)	0.0205 (13)	0.0048 (11)	0.0048 (11)	0.0070 (11)
N10	0.0196 (13)	0.0203 (13)	0.0206 (13)	0.0054 (10)	0.0033 (10)	0.0029 (11)
N11	0.0217 (13)	0.0240 (13)	0.0172 (13)	0.0037 (11)	0.0059 (10)	0.0012 (10)
N12	0.0273 (15)	0.0239 (14)	0.0193 (14)	−0.0075 (11)	0.0020 (11)	−0.0079 (11)
C1	0.0257 (17)	0.0386 (19)	0.0260 (17)	0.0113 (15)	0.0110 (14)	0.0111 (15)
C2	0.0212 (15)	0.0205 (15)	0.0180 (15)	0.0050 (12)	0.0011 (12)	0.0036 (12)
C3	0.0204 (15)	0.0189 (14)	0.0169 (15)	0.0054 (12)	0.0011 (12)	0.0011 (12)
C4	0.0187 (15)	0.0227 (15)	0.0230 (16)	0.0110 (12)	0.0063 (12)	0.0074 (13)
C5	0.0148 (14)	0.0225 (15)	0.0265 (16)	0.0034 (12)	0.0041 (12)	0.0125 (13)
C6	0.0221 (16)	0.0212 (15)	0.0148 (15)	0.0014 (12)	0.0024 (12)	0.0003 (12)
C7	0.0194 (16)	0.0245 (15)	0.0153 (15)	0.0025 (12)	0.0023 (12)	0.0018 (12)
C8	0.0263 (17)	0.0353 (18)	0.0307 (18)	0.0078 (14)	0.0070 (14)	0.0217 (15)
C9	0.0279 (17)	0.0238 (16)	0.0290 (18)	0.0020 (13)	0.0129 (14)	0.0052 (14)
C10	0.0240 (16)	0.0233 (15)	0.0147 (14)	0.0075 (12)	0.0069 (12)	0.0034 (12)
C11	0.0246 (16)	0.0223 (15)	0.0190 (15)	0.0064 (13)	0.0062 (13)	0.0023 (12)
C12	0.0239 (16)	0.0196 (15)	0.0242 (16)	0.0021 (12)	0.0050 (13)	0.0032 (13)
S1	0.0218 (4)	0.0219 (4)	0.0222 (4)	0.0040 (3)	0.0031 (3)	0.0076 (3)
O1	0.0187 (11)	0.0242 (11)	0.0227 (11)	0.0010 (9)	0.0035 (9)	0.0088 (9)
O2	0.0221 (11)	0.0241 (11)	0.0225 (11)	0.0046 (9)	0.0091 (9)	0.0108 (9)
O3	0.0239 (11)	0.0239 (11)	0.0273 (12)	0.0080 (9)	0.0003 (9)	0.0169 (9)
C13	0.0220 (15)	0.0191 (15)	0.0184 (15)	0.0072 (12)	0.0050 (12)	0.0002 (12)
C14	0.0200 (16)	0.0253 (16)	0.0215 (16)	0.0072 (13)	0.0035 (12)	0.0032 (13)
C15	0.0238 (16)	0.0169 (14)	0.0246 (16)	0.0019 (12)	0.0058 (13)	0.0055 (12)
C16	0.0195 (15)	0.0212 (15)	0.0207 (15)	0.0032 (12)	0.0002 (12)	0.0028 (12)
C17	0.0237 (16)	0.0220 (15)	0.0225 (16)	0.0017 (13)	0.0042 (13)	0.0051 (13)
Cl1	0.0314 (9)	0.0308 (8)	0.0284 (9)	−0.0015 (7)	0.0048 (7)	0.0074 (7)
Cl2	0.0182 (7)	0.0195 (7)	0.0128 (6)	0.0016 (5)	0.0015 (5)	0.0050 (5)
Cl3	0.0226 (7)	0.0253 (8)	0.0287 (8)	0.0041 (6)	0.0114 (6)	0.0115 (6)
Cl4	0.0133 (6)	0.0211 (7)	0.0223 (7)	0.0067 (5)	0.0008 (5)	0.0155 (6)

### Geometric parameters (Å, º)

**Table:**

Co1—O2W	2.053 (2)	N12—H12A	0.9100
Co1—O1W	2.084 (2)	N12—H12B	0.9100
Co1—O3W	2.092 (2)	N12—H12C	0.9100
Co1—N5	2.146 (3)	C1—C2	1.490 (4)
Co1—N9	2.156 (3)	C1—H1D	0.9800
Co1—N1	2.162 (3)	C1—H1E	0.9800
Co2—N2^i^	2.132 (3)	C1—H1F	0.9800
Co2—N2	2.132 (3)	C3—C4	1.501 (4)
Co2—N10	2.151 (2)	C4—H4D	0.9800
Co2—N10^i^	2.151 (2)	C4—H4E	0.9800
Co2—N6^i^	2.201 (3)	C4—H4F	0.9800
Co2—N6	2.201 (3)	C5—C6	1.502 (4)
O1W—H1X	0.9796	C5—H5D	0.9800
O1W—H1Y	0.9802	C5—H5E	0.9800
O2W—H2X	0.9801	C5—H5F	0.9800
O2W—H2Y	0.9797	C7—C8	1.494 (4)
O3W—H3X	0.9793	C8—H8D	0.9800
O3W—H3Y	0.9807	C8—H8E	0.9800
N1—C2	1.325 (4)	C8—H8F	0.9800
N1—N2	1.404 (4)	C9—C10	1.485 (4)
N2—C3	1.312 (4)	C9—H9A	0.9800
N3—C2	1.346 (4)	C9—H9B	0.9800
N3—C3	1.350 (4)	C9—H9C	0.9800
N3—N4	1.423 (3)	C11—C12	1.492 (4)
N4—H4A	0.9100	C12—H12D	0.9800
N4—H4B	0.9100	C12—H12E	0.9800
N4—H4C	0.9100	C12—H12F	0.9800
N5—C6	1.308 (4)	S1—O3	1.445 (2)
N5—N6	1.407 (3)	S1—O1	1.462 (2)
N6—C7	1.308 (4)	S1—O2	1.464 (2)
N7—C6	1.358 (4)	S1—C15	1.781 (3)
N7—C7	1.366 (4)	C13—C17^ii^	1.377 (4)
N7—N8	1.409 (3)	C13—C14	1.398 (4)
N8—H8A	0.9100	C13—H13	0.9500
N8—H8B	0.9100	C14—C15	1.365 (4)
N8—H8C	0.9100	C14—H14	0.9500
N9—C10	1.319 (4)	C15—C16	1.444 (4)
N9—N10	1.391 (3)	C16—C17	1.417 (5)
N10—C11	1.302 (4)	C16—C16^ii^	1.425 (6)
N11—C10	1.359 (4)	C17—C13^ii^	1.377 (4)
N11—C11	1.369 (4)	C17—H17	0.9500
N11—N12	1.416 (3)		
			
O2W—Co1—O1W	84.31 (9)	H12A—N12—H12B	109.5
O2W—Co1—O3W	86.35 (9)	N11—N12—H12C	109.5
O1W—Co1—O3W	86.73 (9)	H12A—N12—H12C	109.5
O2W—Co1—N5	90.77 (9)	H12B—N12—H12C	109.5
O1W—Co1—N5	89.79 (9)	C2—C1—H1D	109.5
O3W—Co1—N5	175.68 (9)	C2—C1—H1E	109.5
O2W—Co1—N9	94.08 (9)	H1D—C1—H1E	109.5
O1W—Co1—N9	176.90 (9)	C2—C1—H1F	109.5
O3W—Co1—N9	90.53 (9)	H1D—C1—H1F	109.5
N5—Co1—N9	92.89 (10)	H1E—C1—H1F	109.5
O2W—Co1—N1	171.49 (10)	N1—C2—N3	108.6 (3)
O1W—Co1—N1	87.76 (9)	N1—C2—C1	128.7 (3)
O3W—Co1—N1	90.13 (9)	N3—C2—C1	122.7 (3)
N5—Co1—N1	92.27 (9)	N2—C3—N3	108.8 (3)
N9—Co1—N1	93.70 (10)	N2—C3—C4	130.0 (3)
N2^i^—Co2—N2	180.00 (11)	N3—C3—C4	121.1 (3)
N2^i^—Co2—N10	87.58 (9)	C3—C4—H4D	109.5
N2—Co2—N10	92.42 (10)	C3—C4—H4E	109.5
N2^i^—Co2—N10^i^	92.42 (10)	H4D—C4—H4E	109.5
N2—Co2—N10^i^	87.58 (9)	C3—C4—H4F	109.5
N10—Co2—N10^i^	180.0	H4D—C4—H4F	109.5
N2^i^—Co2—N6^i^	93.28 (9)	H4E—C4—H4F	109.5
N2—Co2—N6^i^	86.72 (9)	C6—C5—H5D	109.5
N10—Co2—N6^i^	87.17 (9)	C6—C5—H5E	109.5
N10^i^—Co2—N6^i^	92.83 (9)	H5D—C5—H5E	109.5
N2^i^—Co2—N6	86.72 (9)	C6—C5—H5F	109.5
N2—Co2—N6	93.28 (9)	H5D—C5—H5F	109.5
N10—Co2—N6	92.83 (9)	H5E—C5—H5F	109.5
N10^i^—Co2—N6	87.17 (9)	N5—C6—N7	109.2 (3)
N6^i^—Co2—N6	180.000 (1)	N5—C6—C5	130.4 (3)
Co1—O1W—H1X	109.4	N7—C6—C5	120.4 (3)
Co1—O1W—H1Y	109.6	N6—C7—N7	108.7 (3)
H1X—O1W—H1Y	109.5	N6—C7—C8	129.9 (3)
Co1—O2W—H2X	109.4	N7—C7—C8	121.4 (3)
Co1—O2W—H2Y	109.5	C7—C8—H8D	109.5
H2X—O2W—H2Y	141.1	C7—C8—H8E	109.5
Co1—O3W—H3X	109.3	H8D—C8—H8E	109.5
Co1—O3W—H3Y	109.6	C7—C8—H8F	109.5
H3X—O3W—H3Y	141.1	H8D—C8—H8F	109.5
C2—N1—N2	106.9 (2)	H8E—C8—H8F	109.5
C2—N1—Co1	130.1 (2)	C10—C9—H9A	109.5
N2—N1—Co1	123.00 (18)	C10—C9—H9B	109.5
C3—N2—N1	107.4 (2)	H9A—C9—H9B	109.5
C3—N2—Co2	129.0 (2)	C10—C9—H9C	109.5
N1—N2—Co2	123.60 (18)	H9A—C9—H9C	109.5
C2—N3—C3	108.1 (2)	H9B—C9—H9C	109.5
C2—N3—N4	123.8 (2)	N9—C10—N11	108.1 (3)
C3—N3—N4	128.1 (2)	N9—C10—C9	129.2 (3)
N3—N4—H4A	109.5	N11—C10—C9	122.8 (3)
N3—N4—H4B	109.5	N10—C11—N11	108.2 (3)
H4A—N4—H4B	109.5	N10—C11—C12	131.1 (3)
N3—N4—H4C	109.5	N11—C11—C12	120.7 (3)
H4A—N4—H4C	109.5	C11—C12—H12D	109.5
H4B—N4—H4C	109.5	C11—C12—H12E	109.5
C6—N5—N6	107.3 (2)	H12D—C12—H12E	109.5
C6—N5—Co1	129.4 (2)	C11—C12—H12F	109.5
N6—N5—Co1	123.37 (18)	H12D—C12—H12F	109.5
C7—N6—N5	107.7 (2)	H12E—C12—H12F	109.5
C7—N6—Co2	130.1 (2)	O3—S1—O1	112.19 (13)
N5—N6—Co2	122.12 (18)	O3—S1—O2	113.46 (13)
C6—N7—C7	107.2 (2)	O1—S1—O2	112.75 (13)
C6—N7—N8	128.9 (3)	O3—S1—C15	106.52 (14)
C7—N7—N8	123.9 (2)	O1—S1—C15	105.66 (13)
N7—N8—H8A	109.5	O2—S1—C15	105.50 (14)
N7—N8—H8B	109.5	C17^ii^—C13—C14	120.3 (3)
H8A—N8—H8B	109.5	C17^ii^—C13—H13	119.9
N7—N8—H8C	109.5	C14—C13—H13	119.9
H8A—N8—H8C	109.5	C15—C14—C13	121.1 (3)
H8B—N8—H8C	109.5	C15—C14—H14	119.5
C10—N9—N10	107.7 (2)	C13—C14—H14	119.5
C10—N9—Co1	129.0 (2)	C14—C15—C16	120.7 (3)
N10—N9—Co1	123.31 (18)	C14—C15—S1	118.5 (2)
C11—N10—N9	108.3 (2)	C16—C15—S1	120.8 (2)
C11—N10—Co2	127.8 (2)	C17—C16—C16^ii^	119.7 (4)
N9—N10—Co2	123.28 (18)	C17—C16—C15	122.7 (3)
C10—N11—C11	107.6 (2)	C16^ii^—C16—C15	117.6 (4)
C10—N11—N12	123.8 (3)	C13^ii^—C17—C16	120.6 (3)
C11—N11—N12	128.5 (3)	C13^ii^—C17—H17	119.7
N11—N12—H12A	109.5	C16—C17—H17	119.7
N11—N12—H12B	109.5		
			
O1W—Co1—N1—C2	42.1 (3)	Co1—N1—C2—N3	179.84 (19)
O3W—Co1—N1—C2	−44.6 (3)	N2—N1—C2—C1	179.3 (3)
N5—Co1—N1—C2	131.8 (3)	Co1—N1—C2—C1	−1.0 (5)
N9—Co1—N1—C2	−135.2 (3)	C3—N3—C2—N1	−0.7 (3)
O1W—Co1—N1—N2	−138.2 (2)	N4—N3—C2—N1	−179.8 (3)
O3W—Co1—N1—N2	135.1 (2)	C3—N3—C2—C1	−180.0 (3)
N5—Co1—N1—N2	−48.5 (2)	N4—N3—C2—C1	0.9 (4)
N9—Co1—N1—N2	44.5 (2)	N1—N2—C3—N3	−1.0 (3)
C2—N1—N2—C3	0.6 (3)	Co2—N2—C3—N3	178.92 (19)
Co1—N1—N2—C3	−179.2 (2)	N1—N2—C3—C4	178.6 (3)
C2—N1—N2—Co2	−179.37 (19)	Co2—N2—C3—C4	−1.4 (5)
Co1—N1—N2—Co2	0.9 (3)	C2—N3—C3—N2	1.1 (3)
N10—Co2—N2—C3	132.7 (3)	N4—N3—C3—N2	−179.8 (3)
N10^i^—Co2—N2—C3	−47.3 (3)	C2—N3—C3—C4	−178.6 (3)
N6^i^—Co2—N2—C3	45.7 (3)	N4—N3—C3—C4	0.5 (5)
N6—Co2—N2—C3	−134.3 (3)	N6—N5—C6—N7	−1.0 (3)
N10—Co2—N2—N1	−47.3 (2)	Co1—N5—C6—N7	−179.58 (19)
N10^i^—Co2—N2—N1	132.7 (2)	N6—N5—C6—C5	179.7 (3)
N6^i^—Co2—N2—N1	−134.3 (2)	Co1—N5—C6—C5	1.1 (5)
N6—Co2—N2—N1	45.7 (2)	C7—N7—C6—N5	0.9 (3)
O2W—Co1—N5—C6	40.3 (3)	N8—N7—C6—N5	−179.4 (3)
O1W—Co1—N5—C6	−44.0 (3)	C7—N7—C6—C5	−179.7 (3)
N9—Co1—N5—C6	134.5 (3)	N5—N6—C7—N7	−0.2 (3)
N1—Co1—N5—C6	−131.7 (3)	Co2—N6—C7—N7	−175.57 (19)
O2W—Co1—N5—N6	−138.0 (2)	N5—N6—C7—C8	−177.4 (3)
O1W—Co1—N5—N6	137.7 (2)	Co2—N6—C7—C8	7.3 (5)
N9—Co1—N5—N6	−43.9 (2)	C6—N7—C7—N6	−0.4 (3)
N1—Co1—N5—N6	49.9 (2)	N8—N7—C7—N6	179.9 (3)
C6—N5—N6—C7	0.8 (3)	C6—N7—C7—C8	177.1 (3)
Co1—N5—N6—C7	179.4 (2)	N8—N7—C7—C8	−2.7 (5)
C6—N5—N6—Co2	176.57 (19)	N10—N9—C10—N11	−1.1 (3)
Co1—N5—N6—Co2	−4.8 (3)	Co1—N9—C10—N11	177.3 (2)
N2^i^—Co2—N6—C7	−48.8 (3)	N10—N9—C10—C9	178.4 (3)
N2—Co2—N6—C7	131.2 (3)	Co1—N9—C10—C9	−3.2 (5)
N10—Co2—N6—C7	−136.2 (3)	C11—N11—C10—N9	1.9 (4)
N10^i^—Co2—N6—C7	43.8 (3)	N12—N11—C10—N9	−178.9 (3)
N2^i^—Co2—N6—N5	136.4 (2)	C11—N11—C10—C9	−177.7 (3)
N2—Co2—N6—N5	−43.6 (2)	N12—N11—C10—C9	1.5 (5)
N10—Co2—N6—N5	49.0 (2)	N9—N10—C11—N11	1.3 (3)
N10^i^—Co2—N6—N5	−131.0 (2)	Co2—N10—C11—N11	−169.8 (2)
O2W—Co1—N9—C10	−35.7 (3)	N9—N10—C11—C12	−176.3 (3)
O3W—Co1—N9—C10	50.7 (3)	Co2—N10—C11—C12	12.5 (5)
N5—Co1—N9—C10	−126.7 (3)	C10—N11—C11—N10	−2.0 (4)
N1—Co1—N9—C10	140.9 (3)	N12—N11—C11—N10	178.9 (3)
O2W—Co1—N9—N10	142.5 (2)	C10—N11—C11—C12	175.9 (3)
O3W—Co1—N9—N10	−131.1 (2)	N12—N11—C11—C12	−3.2 (5)
N5—Co1—N9—N10	51.5 (2)	C17^ii^—C13—C14—C15	−0.3 (5)
N1—Co1—N9—N10	−41.0 (2)	C13—C14—C15—C16	−0.7 (5)
C10—N9—N10—C11	−0.2 (3)	C13—C14—C15—S1	179.1 (2)
Co1—N9—N10—C11	−178.7 (2)	O3—S1—C15—C14	−1.7 (3)
C10—N9—N10—Co2	171.5 (2)	O1—S1—C15—C14	117.8 (3)
Co1—N9—N10—Co2	−7.0 (3)	O2—S1—C15—C14	−122.6 (3)
N2^i^—Co2—N10—C11	41.0 (3)	O3—S1—C15—C16	178.2 (2)
N2—Co2—N10—C11	−139.0 (3)	O1—S1—C15—C16	−62.3 (3)
N6^i^—Co2—N10—C11	−52.4 (3)	O2—S1—C15—C16	57.3 (3)
N6—Co2—N10—C11	127.6 (3)	C14—C15—C16—C17	−179.7 (3)
N2^i^—Co2—N10—N9	−128.9 (2)	S1—C15—C16—C17	0.4 (4)
N2—Co2—N10—N9	51.1 (2)	C14—C15—C16—C16^ii^	1.4 (5)
N6^i^—Co2—N10—N9	137.7 (2)	S1—C15—C16—C16^ii^	−178.5 (3)
N6—Co2—N10—N9	−42.3 (2)	C16^ii^—C16—C17—C13^ii^	−0.1 (5)
N2—N1—C2—N3	0.1 (3)	C15—C16—C17—C13^ii^	−179.0 (3)

Symmetry codes: (i) −*x*+1, −*y*+1, −*z*+1; (ii) −*x*+2, −*y*, −*z*+2.

### Hydrogen-bond geometry (Å, º)

**Table:**

*D*—H···*A*	*D*—H	H···*A*	*D*···*A*	*D*—H···*A*
O1*W*—H1*X*···Cl3^i^	0.98	2.06	2.765 (3)	127
O1*W*—H1*Y*···Cl3^iii^	0.98	1.89	2.701 (3)	138
O2*W*—H2*X*···Cl1	0.98	2.28	2.929 (3)	122
O2*W*—H2*Y*···Cl3^iii^	0.98	2.82	3.749 (3)	158
O3*W*—H3*X*···O2^iv^	0.98	1.89	2.773 (3)	148
O3*W*—H3*Y*···Cl4^iv^	0.98	2.18	3.114 (3)	159
N8—H8*A*···O3^i^	0.91	2.21	3.009 (3)	146

Symmetry codes: (i) −*x*+1, −*y*+1, −*z*+1; (iii) *x*, *y*, *z*−1; (iv) −*x*+2, −*y*+1, −*z*+1.

## References

[bb1] Brandenburg, K. (1999). *DIAMOND* Crystal Impact GbR, Bonn, Germany.

[bb2] Bruker (2001). *SADABS* Bruker AXS Inc., Madison, Wisconsin, USA.

[bb3] Bruker (2007). *SMART* and *SAINT* Bruker AXS Inc., Madison, Wisconsin, USA.

[bb4] Sheldrick, G. M. (2008). *Acta Cryst.* A**64**, 112–122. 10.1107/S0108767307043930 18156677

[bb5] Tong, Y.-Z., Wang, Q.-L., Si, M., Qi, J., Yan, S.-P., Yang, G.-M., Cheng, P. & Liao, D.-Z. (2011). *Polyhedron*, **30**, 3151–3157.

